# The prevalence of antibodies to SARS-CoV-2 among blood donors in China

**DOI:** 10.1038/s41467-021-21503-x

**Published:** 2021-03-02

**Authors:** Le Chang, Wangheng Hou, Lei Zhao, Yali Zhang, Yanbin Wang, Linfeng Wu, Tingting Xu, Lilin Wang, Juan Wang, Jian Ma, Lan Wang, Junpeng Zhao, Jing Xu, Juan Dong, Ying Yan, Ru Yang, Yu Li, Fei Guo, Wenjuan Cheng, Yingying Su, Jinfeng Zeng, Wei Han, Tong Cheng, Jun Zhang, Quan Yuan, Ningshao Xia, Lunan Wang

**Affiliations:** 1 National Center for Clinical Laboratories, Beijing Hospital, National Center of Gerontology; Institute of Geriatric Medicine, Chinese Academy of Medical Sciences, Beijing, P.R. China; 2 Beijing Engineering Research Center of Laboratory Medicine, Beijing Hospital, Beijing, P.R. China; 3 State Key Laboratory of Molecular Vaccinology and Molecular Diagnostics, School of Public Health, Xiamen University, Xiamen, P.R. China; 4 National Institute of Diagnostics and Vaccine Development in Infectious Diseases, School of Public Health & School of Life Science, Xiamen University, Xiamen, P.R. China; 5 Department of Laboratory, Wuhan Blood Center, Wuhan, P.R. China; 6 Confirmation Laboratory for Transfusion Transmitted Disease, Institute of Blood Transfusion of Hubei Province, Wuhan, P.R. China; 7 Blood Screening Laboratory, Hebei Province Blood Center, Shijiazhuang, P.R. China; 8 Shenzhen Blood Center, Shenzhen, P.R. China; 9 Wuhan Blood Center, Wuhan, P.R. China; 10 Department of Transfusion Research, Wuhan Blood Center, Wuhan, P.R. China; 11 Graduate School, Peking Union Medical College, Chinese Academy of Medical Sciences, Beijing, P.R. China

**Keywords:** SARS-CoV-2, Viral infection, Epidemiology, Risk factors

## Abstract

In this study, we investigate the seroprevalence of SARS-CoV-2 antibodies among blood donors in the cities of Wuhan, Shenzhen, and Shijiazhuang in China. From January to April 2020, 38,144 healthy blood donors in the three cities were tested for total antibody against SARS-CoV-2 followed by pseudotype SARS-CoV-2 neutralization tests, IgG, and IgM antibody testing. Finally, a total of 398 donors were confirmed positive. The age- and sex-standardized SARS-CoV-2 seroprevalence among 18–60 year-old adults (18–65 year-old in Shenzhen) was 2.66% (95% CI: 2.24%–3.07%) in Wuhan, 0.033% (95% CI: 0.0029%–0.267%) in Shenzhen, and 0.0028% (95% CI: 0.0001%–0.158%) in Shijiazhuang, respectively. Female sex and older-age were identified to be independent risk factors for SARS-CoV-2 seropositivity among blood donors in Wuhan. As most of the population of China remained uninfected during the early wave of the COVID-19 pandemic, effective public health measures are still certainly required to block viral spread before a vaccine is widely available.

## Abstract (web summary)

Here, the authors determine seroprevalence of antibodies to SARS-CoV-2 in healthy blood donors in the cities of Wuhan, Shenzhen, and Shijiazhuang in China between January and April 2020. The age- and sex-standardized SARS-CoV-2 seroprevalence among 18–60 year-old adults is, with 2.66%, the highest in Wuhan.

## Introduction

Coronavirus disease 2019 (COVID-19), first found in December 2019, fast spread all over the world and was declared as a pandemic in March 2020^1^. The total of confirmed cases has surpassed 70 million, involving 220 countries, and more than 1.5 million people died resulting from acute respiratory diseases and their related complications^2^. A novel beta-coronavirus was identified as the original etiological agent of COVID-19. The genome of the new virus was 70% similar to that of severe acute respiratory syndrome coronavirus (SARS-CoV), and it was designated SARS-CoV-2^3,4^.

Nucleic acid testing of SARS-CoV-2 from the upper or lower respiratory tract, feces, urine, or other specimens could quickly identify infected people from suspected cases. The timely diagnosis could help reduce patient gathering and shorten the length of stay in clinics, thus promoting effective infection control management^5^. However, the COVID-19 pandemic is like an iceberg^6^: what we can see is those who are severe cases, part of mild-to-moderate cases, and known asymptomatic cases diagnosed by the screening of close contacts of COVID-19 cases or random testing of a specific population. An uncertain number of asymptomatic individuals and parts of mild cases may be missed, like the bottom of the iceberg under the water. These asymptomatic individuals may contribute to the transmission of the disease^7,8^. Moreover, it is crucial to identify the asymptomatic infections to estimate the disease burden and to get a better understanding of the real case fatality rate^9,10^. The missing information could be obtained by screening the population for specific antibodies using validated serologic assays.

Host humoral immune response to SARS-CoV-2 among COVID-19-confirmed patients had been characterized by several studies^11–14^. Total antibody (TAb) specific to SARS-CoV-2, which had been demonstrated to be the most sensitive and earliest serologic biomarker, usually increased since the second week of symptom onset^12^, and second week after onset, all infected cases showed reactive results^11^. In contrast, the IgM and IgG seroconversion generally occurred on the second or third week^13^, following a quick decrease of IgM and a longtime IgG persistence^12^. So far, only limited information on serologic screening of specific asymptomatic people showed that seroprevalence of SARS-CoV-2 varied from 1.6% to 4.1% among different countries and populations^15–17^. However, comprehensive data of antibody response against SARS-CoV-2 in asymptomatic individuals of mainland China are unclear.

In this work, we investigated the prevalence of SARS-CoV-2 antibodies among donors who donated their blood from January to April 2020 in the cities of Wuhan, Shenzhen, and Shijiazhuang. These three different cities, locating in the central, south, and north of China, have similar population size but of distinct COVID-19 incidence. Moreover, the potential risk factors for SARS-CoV-2 seropositivity were analyzed.

## Results

### Characteristics of enrolled blood donors

Almost all blood donors donated in the three blood centers during the study period were enrolled, totaling 38,144 blood donors, including 17,794 were from Wuhan, 6810 from Shenzhen, and 13,540 from Shijiazhuang. The characteristics of the involved donors were summarized in Table 1. The median age was 33 (IQR, 19–47), 36 (IQR, 19–53), and 40 (IQR, 33–48) for donors from the three cities, respectively. Among these donors, 29.5–37.7% were female.

**Table 1:** Characteristics of enrolled blood donors.

	Wuhan	Shenzhen	Shijiazhuang
	(*n* = 17,794)	(*n* = 6810)	(*n* = 13,540)
Sex (%)			
Male	11,077 (62.3)	4428 (65.0)	9542 (70.5)
Female	6717 (37.7)	2382 (35.0)	3998 (29.5)
Age (%)			
Median (IQR)	33 (19–47)	36 (19-53)	40 (33–48)
18–25	3891 (21.9)	1330 (19.5)	1098 (8.1)
26–35	6910 (38.8)	2046 (30.0)	3692 (27.3)
36–45	4561 (25.6)	1943 (28.5)	4310 (31.8)
46–55	2318 (13.0)	1326 (19.5)	4032 (29.8)
>55	114 (0.6)	165 (2.4)	408 (3.0)
ABO blood type (%)			
A	5660 (31.8)	2015 (29.6)	3212 (23.7)
B	4462 (25.1)	1701 (25.0)	4765 (35.2)
O	5971 (33.6)	2667 (39.2)	4078 (30.1)
AB	1675 (9.4)	427 (6.3)	1485 (11.0)
Missing data	26 (0.1)	0	0
Rh (D) blood type (%)			
+	17,605 (98.9)	6758 (99.2)	13,483 (99.6)
−	158 (0.9)	52 (0.8)	57 (0.4)
Missing data	31 (0.2)	0	0
Ethnicity (%)			
Han	17,126 (96.2)	6519 (95.7)	13,414 (99.1)
Non-Han	533 (3.0)	274 (4.0)	124 (0.9)
Missing data	135 (0.8)	17 (0.2)	2 (0.0)
Occupation (%)			
Healthcare worker	438 (2.5)	539 (7.9)	812 (6.0)
Civil worker	358 (2.0)	203 (3.0)	412 (3.0)
Teacher	490 (2.8)	68 (1.0)	196 (1.4)
Student	1457 (8.2)	307 (4.5)	390 (2.9)
Office worker	4576 (25.7)	2470 (36.3)	1410 (10.4)
Worker	1726 (9.7)	613 (9.0)	1097 (8.1)
Military personnel	101 (0.6)	43 (0.6)	42 (0.3)
Farmer	353 (2.0)	90 (1.3)	2614 (19.3)
Others	6931 (39.0)	2476 (36.4)	6546 (48.3)
Missing data	1364 (7.7)	1 (0.0)	21 (0.2)
Education level (%)			
Master/doctorate	968 (5.4)	343 (5.0)	89 (0.7)
Bachelor	5323 (29.9)	1806 (26.5)	1669 (12.3)
college	4779 (26.9)	1516 (22.3)	1916 (14.2)
High school	2981 (16.8)	1472 (21.6)	4125 (30.5)
lower than high school	1451 (8.2)	1641(24.1)	5734 (42.3)
Missing data	2292(12.9)	32 (0.5)	7(0.0)

### Antibody prevalence of SARS-CoV-2 among blood donors

**Fig. 1:**
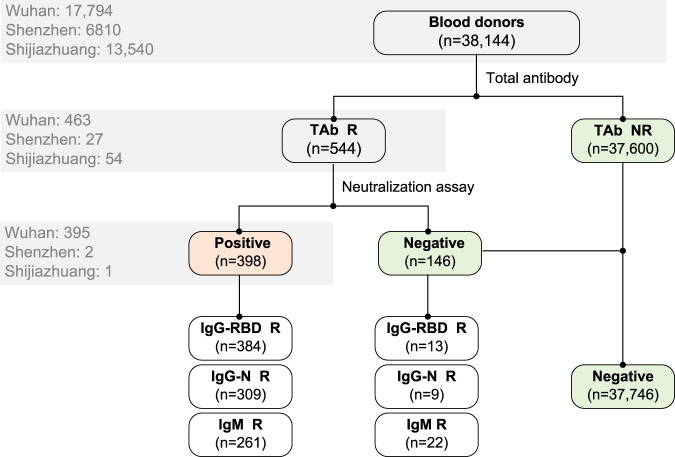
Flowchart of screening and confirmatory procedure. A total of 38,144 blood donations were tested for SARS-CoV-2 total antibody (TAb). Those reactive were further confirmed by neutralization assay and tested for IgG antibody against receptor-binding domain (RBD) of the spike protein and nucleoprotein of SARS-CoV-2 (IgG-RBD and IgG-N), and IgM antibody against SARS-CoV-2. Three-hundred ninety-eight donors were finally confirmed for SARS-CoV-2 seropositivity. The numbers of donations tested for specific antibodies in three cities are shown in the gray box. The crude seroprevalence of antibodies to SARS-CoV-2 among blood donors was calculated by the number of confirmed positive donors divided by the total number of tested donors: 2.22% (395/17,794, 95% CI: 2.01–2.45%) in Wuhan, 0.029% (2/6810, 95% CI: 0.0081–0.11%) in Shenzhen, and 0.0074% (1/13,540, 95% CI: 0.0013–0.042%) in Shijiazhuang, respectively. R, reactive; NR, nonreactive; IgG-RBD, IgG antibody against receptor-binding domain (RBD) of the spike protein of SARS-CoV-2; IgG-N, IgG antibody against nucleoprotein of SARS-CoV-2.

Among all samples, 544 (544/38,144, 1.43%) were TAb-positive (all were HIV-free), of which 463 (463/17,794, 2.60%) were from Wuhan, 27 (27/6810, 0.396%) from Shenzhen, and 54 (54/13,540, 0.399%) from Shijiazhuang. All the TAb-positive samples were further tested for SARS-CoV-2 IgG antibody against receptor-binding domain (IgG-RBD) of the spike protein or nucleoprotein (IgG-N), IgM antibody, and pseudotype lentivirus-based neutralization tests (ppNAT). Finally, 398 (398/463, 85.96%) donors confirmed the presence of SARS-CoV-2 neutralization antibody by the ppNAT tests. The screening and confirmatory procedures were shown in Fig. 1. The results revealed that the crude SARS-CoV-2 seroprevalence in the three different populations was 2.22% (395/17,794, 95% CI: 2.01–2.45%) in Wuhan, 0.029% (2/6,810, 95% CI: 0.0081–0.11%) in Shenzhen, and 0.0074% (1/13,540, 95% CI: 0.0013–0.042%) in Shijiazhuang, respectively. After age–sex standardization by population distribution of the three cities, the SARS-CoV-2 seroprevalence among 18–60-year-old adults (18–65-year-old adults in Shenzhen) was 2.66% (95% CI: 2.24–3.07%) in Wuhan, 0.033% (95% CI: 0.0029–0.267%) in Shenzhen, and 0.0028% (95% CI: 0.0001–0.158%) in Shijiazhuang, respectively. Among all confirmed seropositive donors, 96.48% (384/398) were IgG-RBD positive, 77.64% (309/398) were IgG-N positive, and IgM antibody was detectable in 261 (65.58%, 261/398) donors.

**Fig. 2:**
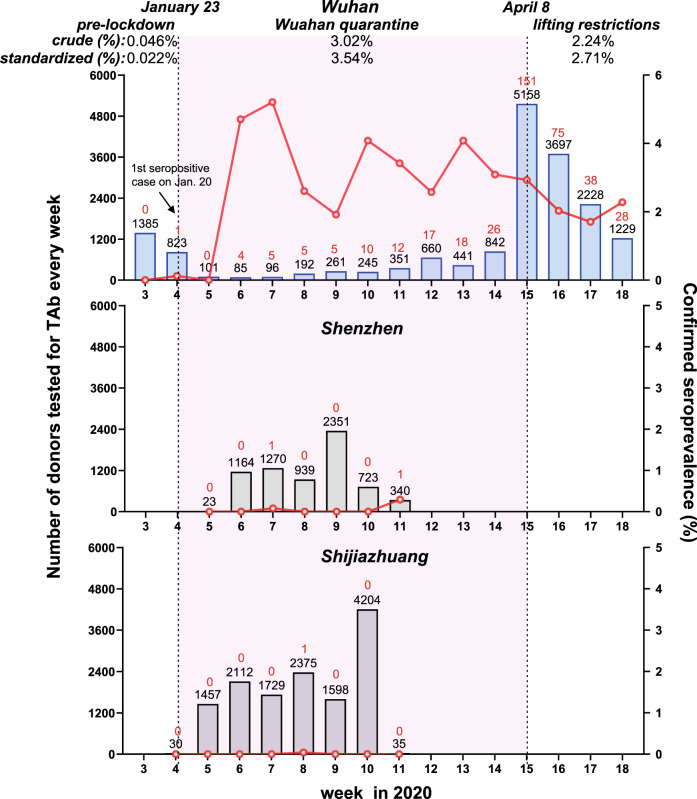
Weekly seroprevalence of SARS-CoV-2 antibody during different periods from January to April 2020 in the cities of Wuhan, Shenzhen, and Shijiazhuang. The number of donors tested for total antibody (TAb) every week (the black numbers on the top of each histogram) is shown in histograms. The number of confirmed positive cases is shown in red numbers on the top of each histogram. The confirmed seropositive rate (number of confirmed positive donors/number of donors tested for TAb) in each week is shown in red lines. The first donor confirmed positive by the pseudotype lentivirus-based neutralization tests in Wuhan was donated on January 20, the fourth week of 2020. Lockdown of Wuhan City started on January 23 and on April 8, all the travel restrictions in Wuhan were lifted. The period of study in Wuhan is divided into three stages: prelockdown (Jan 15–Jan 22), lockdown (Jan 23–Apr 7), and lifting restrictions (Apr 8–Apr 30). The confirmed seroprevalences of the three stages varied: only one from 2164 donors was confirmed in the first stage (0.046%, 95% CI: 0.082–0.26%); 169 donors with confirmed serological evidence were identified from 5587 donors, suggesting a seroprevalence of 3.02% (95% CI: 2.60–3.51%) in the lockdown stage. After April 8, we further tested a total of 10,043 donors, and found out that 225 were confirmed SARS-CoV-2 seropositive (2.24%, 95% CI: 1.97–2.55%). The peak of seroprevalence (5.21%, 5/96) occurred in the stage of lockdown. The seroprevalence of the three stages, after age–sex standardization with the population distribution in the city of Wuhan of 18–60-year-old adults, was 0.022% (95% CI: 0.005–1.494%), 3.54% (95% CI: 2.71–4.37%), and 2.71% (95% CI: 2.09–3.32%), respectively.

In samples from Wuhan involved in our study, there were 2164 blood donations that were collected before January 23, 2020 when Wuhan was quarantined, including 1385 samples were donated from January 15 to 18 (week 3 of 2020) and 779 samples were donated during January 19 to 22. Among these donations, only one was confirmed for SARS-CoV-2 seropositivity, which was donated on January 20, 2020. The data suggested that the SARS-CoV-2 seroprevalence in the blood donor population of Wuhan from mid-to-late January 2020 was about 0.046% (1/2,164, 95% CI: 0.082–0.26%). During the Wuhan quarantine period (from January 23 to April 7), 169 donors were identified from 5587 Wuhan’s blood donors, suggesting a seroprevalence of 3.02% (169/5,587, 95% CI: 2.60–3.51%). After April 8, when the city was eased (from April 8 to April 30), we further tested a total of 10,043 donors and found out that 225 donors were confirmed SARS-CoV-2 seropositive. The SARS-CoV-2 seroprevalence among Wuhan’s blood donors during this period was 2.24% (225/10,043, 95% CI: 1.97–2.55%). In a time-series analysis (Fig. 2, upper panel), the highest seroprevalence (5.21%, 5/96, 95% CI: 2.25–11.62%) was observed among Wuhan’s blood donors at the 7th week of 2020 (from February 9 to 15), about 3 weeks after the quarantine. Since then, the seropositive rate ceased to increase in blood donors of Wuhan. Given consideration of age and gender differences of the donors’ population during the three different stages (Fig. S1 in the Supplementary Information), the seroprevalence of the three stages after age–sex standardization (18–60-year-old adults) was 0.022% (95% CI: 0.005–1.494%), 3.54% (95% CI: 2.71–4.37%), and 2.71% (95% CI: 2.09–3.32%), respectively. In comparison to Wuhan, the SARS-CoV-2 seroprevalence among donors of Shenzhen and Shijiazhuang was very low during the Wuhan quarantine period (Fig. 2, middle and lower panels).

### Associations between the titer of ppNAT and other antibody markers

**Fig. 3:**
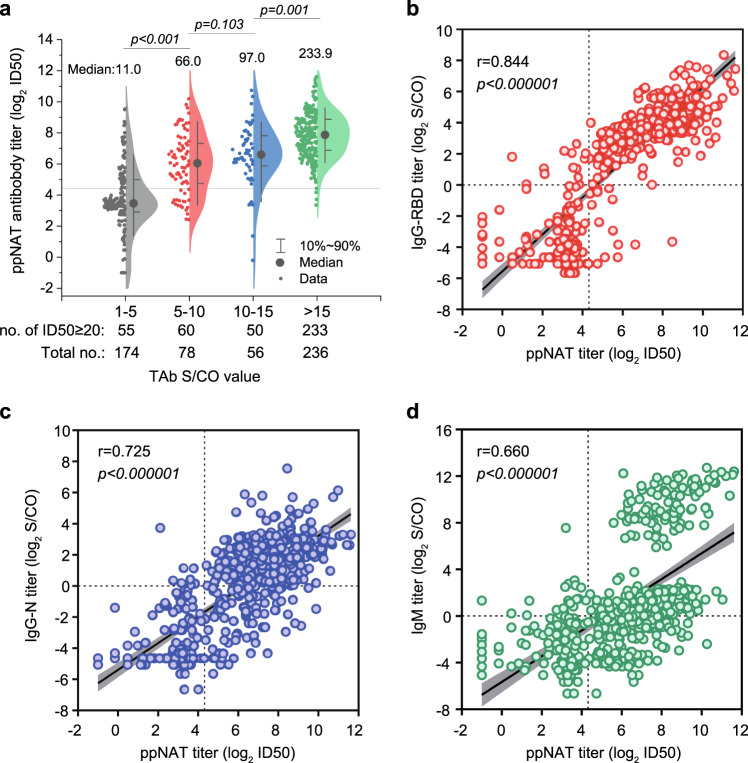
Relationships between ppNAT titer and ELISA-binding titers among all TAb-positive samples. **a** The relationship between ID50 (maximal dilution ratio at half-infection inhibition) of pseudotype lentivirus-based neutralization tests (ppNAT) and different groups of signal to the cutoff ratio (S/CO) of total antibody (TAb). The horizontal gray line shows the cutoff value of ppNAT (ID50 = 20). The neutralization titer successively elevated with increasing S/CO of TAb among all samples (*p* < 0.001, Group 1–5 vs. 5–10: *p* < 0.001; Group 1–5 vs. 10–15: *p* < 0.001; Group 1–5 vs. >15: *p* < 0.001; Group 5–10 vs. 10–15: *p* = 0.103; Group 5–10 vs. >15: *p* < 0.001; Group 10–15 vs. >15: *p* = 0.001). Median titers of the four groups are 11.0, 66.0, 97.0, and 233.9, respectively. **b** The relationship between ppNAT ID50 titer and titer of IgG antibody against receptor-binding domain (RBD) of the spike protein of SARS-CoV-2 (IgG-RBD). The correlation coefficient (*r*) by Spearman rank correlation analysis between IgG-RBD and ID50 was as high as 0.844 (*p* < 0.000001). The dotted lines show the cutoff value of ppNAT (ID50 = 20) and IgG-RBD assay (S/CO = 1.0). **c** The relationship between ppNAT ID50 titer and titer of IgG antibody against nucleoprotein of SARS-CoV-2 (IgG-N). The correlation coefficient (*r*) between IgG-N and ID50 was 0.725 (*p* < 0.000001). The dotted lines show the cutoff value of ppNAT (ID50 = 20) and IgG-N assay (S/CO = 1.0). **d** The relationship between ppNAT ID50 titer and titer of IgM antibody against SARS-CoV-2. The correlation coefficient (*r*) between IgM and ID50 is 0.660 (*p* < 0.000001). The dotted lines show the cutoff value of ppNAT (ID50 = 20) and IgM assay (S/CO = 1.0). *p*-values by Kruskal–Wallis test followed by Bonferroni’s multiple-comparison post hoc test (two-sided) are indicated in (**a**), and the Spearman correlation coefficient (*r*) and *p*-values by Spearman’s test are indicated in (**b**–**d**). The error band in gray shows the 95% confidence intervals of the fitted line by Spearman rank correlation analysis in (**b**–**d**).

We analyzed the associations between the titer of ppNAT (maximal dilution ratio at half-infection inhibition, ID50) with TAb (undiluted S/CO), IgG-RBD (dilution-quantitative S/CO), IgG-N (dilution- quantitative S/CO), and IgM (dilution-quantitative S/CO) among all 544 donations with detectable TAb. As the results show in Fig. 3a, the median ppNAT titer successively elevated with the increase of TAb S/CO value. Notably, the percentage of samples with a ppNAT ID50 ≥ 20 (confirmatory presence of neutralization antibody) was 31.61% (55/174), 76.92% (60/78), 89.29% (50/56), and 98.73% (233/236) among those with TAb S/CO strata of 1–5, 5–10, 10–15, and >15, respectively. Moreover, the ppNAT titer was positively correlated with the titers of IgG-RBD (*r* = 0.844, *p* < 0.000001, Fig. 3b), IgG-N (*r* = 0.725, *p* < 0.000001, Fig. 3c), and IgM (*r* = 0.660, *p* < 0.000001, Fig. 3d), respectively. Overall, the IgG-RBD titer showed the best correlation with the confirmatory ppNAT titer among TAb-positive samples. The average titer of IgG-N was significantly lower than that of IgG-RBD (*p* < 0.001), suggesting that the antibody response against viral nucleoprotein may be weaker than that to viral spike protein.

Although the high sensitivity and specificity of the double-sandwich RBD-based TAb ELISA had been demonstrated in COVID-19 patients and asymptomatic infections, the positive predictive value (PPV) of this test was significantly varied among different populations when it was used alone. In this study, the PPV of TAb ELISA was 85.31% (395/463), 7.41% (2/27), and 1.85% (1/54) in samples from Wuhan, Shenzhen, and Shijiazhuang, respectively. Overall, the PPV values of TAb in different populations were highly associated with the reported numbers of COVID-19-confirmed cases of these cities. These results highlighted the necessity of the confirmatory testing for TAb-positive samples, in particular for those from the nonepidemic area. To establish an optimized strategy with practical applicability for the situation when a cell-based neutralization test is unavailable, we analyzed the diagnostic performance for the combined use of TAb and other immunoassay-determined antibody markers. Table 2 listed the added diagnosis value of IgG-RBD, IgG-N, and IgM in TAb-positive samples from Wuhan (*n* = 463) and the other two cities (*n* = 81). Apparently, additional IgG-RBD testing in TAb-positive samples was a preferred screening approach, which showed a significantly improved PPV (TAb/IgG-RBD double reactive, PPV: 98.71%) with little missing (12/395, 3.04%) of true-positive samples among blood donors in Wuhan (Table 2). However, this combined strategy showed minimal PPV improvement among donors in Shenzhen and Shijiazhuang (Table 2).

**Table 2:** Comparison of performance of the combined markers in the detection of confirmatory SARS-CoV-2 antibody among blood donors in Wuhan and other cities.

Positive criterion	Screen out positive no.	True-positive no. (PPV, %)	True-positive missing no. (%)
Among TAb(+) Donors in Wuhan (*n* = 463)			
TAb-R	463	395 (85.31%)	0
TAb/IgM DR	268	260(97.01%)	135 (34.18%)
TAb/IgG-RBD DR	388	383 (98.71%)	12 (3.04%)
TAb/IgG-N DR	313	309 (98.72%)	86 (21.77%)
Among TAb(+) Donors in the other two cities (*n* = 81)			
TAb-R	81	3 (3.70 %)	0
TAb/IgM DR	16	3 (18.75%)	0
TAb/IgG-RBD DR	9	1 (11.11%)	2 (66.67%)
TAb/IgG-N DR	5	0	3 (100.00%)

*TAb* total antibody against SARS-CoV-2, *IgG-RBD* IgG antibody against receptor-binding domain (RBD) of the spike protein of SARS-CoV-2, *IgG-N* IgG antibody against nucleoprotein of SARS-CoV-2, *R* reactive, *DR* reactive in double assays, *PPV* positive predictive value.

### Potential risk factors associating with asymptomatic infections

**Fig. 4:**
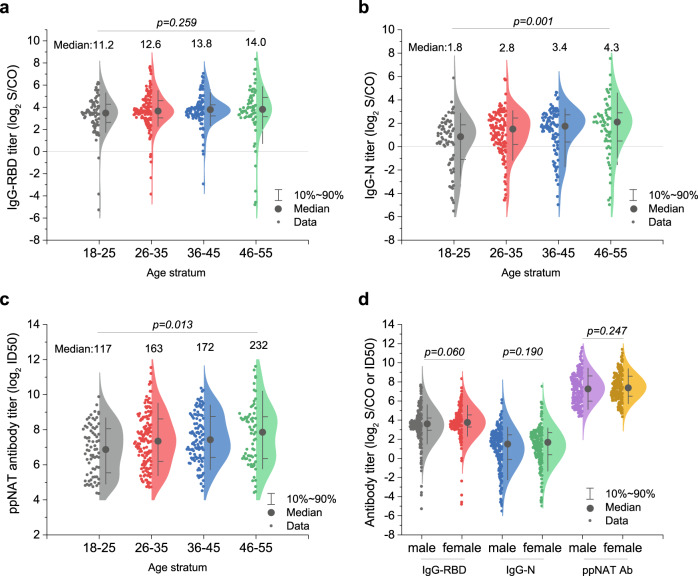
Relationship between specific antibody titer and gender or age. **a** Relationship between the titer of IgG antibody against receptor-binding domain (RBD) of the spike protein of SARS-CoV-2 (IgG-RBD) and four different age groups. No statistical significance is shown among different age groups (*p* = 0.259). The horizontal gray line shows the cutoff value of IgG-RBD assay (S/CO = 1.0). Median titers of the four groups are 11.2, 12.6, 13.8, and 14.0, respectively. **b** Relationship between the titer of IgG antibody against nucleoprotein of SARS-CoV-2 (IgG-N) and four different age groups. A significant difference is shown among different age groups (*p* = 0.001). The IgG-N titer of age group 18–25 is lower than that of group 36–45 (*p* = 0.005) and 46–60 (*p* = 0.001).The horizontal gray line shows the cutoff value of IgG-N assay (S/CO = 1.0). Median titers of the four groups are 1.8, 2.8, 3.4, and 4.3, respectively. **c** Relationship between the titer of ppNAT ID50 (maximal dilution ratio at half-infection inhibition) and four different age groups. A significant difference is shown among different age groups (*p* = 0.013). Median titers of the four groups are 117, 163, 172, and 232, respectively. The ID50 titer of age group 18–25 is lower than that of group 36–45 (*p* = 0.027) and group 46–55 (*p* = 0.022). **d** Relationship between the titer of specific IgG or ID50 of pseudotype lentivirus-based neutralization tests (ppNAT) and gender. There was no difference in the titers of IgG-RBD, IgG-N, or neutralizing antibodies between males and females (*p* = 0.060, 0.190, and 0.247). Median titers of the six groups are 12.2, 13.5, 2.8, 3.2, 153, and 165, respectively. *p*-values by Kruskal–Wallis (two-sided) test are indicated in (**a**), by Kruskal–Wallis test followed by Bonferroni’s multiple-comparison post hoc test (two-sided) in (**b**), and (**c**), and by Mann–Whitney *U* test (two-sided) in (**d**).

As only three confirmed seropositive donors were identified in the cities of Shenzhen and Shijiazhuang, we only performed risk factor analysis among 17,794 donors in Wuhan. As all confirmatory seropositive donors identified in our study declared they never had symptoms of COVID-19, they might experience a past asymptomatic infection. Multivariate regression analysis revealed that age and gender were independent risk factors for the presence of antibodies against SARS-CoV-2 (Table 3). Females showed a 1.8-fold (adjusted odd ratio, OR, 95% CI: 1.5–2.2, *p* < 0.000001) increased risk in comparison with males. Compared with donors with age ranging from 18 to 25, the adjusted OR was 1.1 (95% CI: 0.9–1.5, *p* = 0.392) for donors with age of 26–35, 1.4 (95% CI: 1.0–1.8, *p* = 0.048) for donors with age ranging from 36 to 45, 1.6 (95% CI: 1.2–2.3, *p* = 0.005) for donors with age ranging from 46 to 55, and 4.1 (95% CI: 1.9–8.8, *p* = 0.0003) for donors with an age of 55 or older. No statistical significance was found on donors with different ABO or Rh(D) blood types, ethnicities, occupations, or education levels by single-factor logistic regression analysis (*p* = 0.120, 0.725, 0.861, 0.443, and 0.582, respectively). We further analyzed the relationship between antibody titers and age or gender. The results (Fig. 4) revealed that the median levels of IgG-N (*p* = 0.001) and ppNAT (*p* = 0.013) showed a significant increasing trend with older age, whereas it was not statistically significant for IgG-RBD level (*p* = 0.259). Moreover, no significant difference was observed on the median levels of IgG-RBD, IgG-N, and ppNAT between males and females (*p* = 0.060, 0.190, and 0.247, respectively).

**Table 3:** Binary logistic regression analysis of risk factors for the confirmed seroprevalence among blood donors in Wuhan.

	Confirmed donor no. (seroprevalence %)	OR (95%CI)	*p* value	Adjusted OR (95%CI)	*p* value
Sex					
Male	191 (1.7)	1.0		1.0	–
Female	204 (3.0)	1.8 (1.5–2.2)	<0.000001	1.8 (1.5–2.2)	<0.000001
Age			0.0006		0.0005
Median (IQR)	34 (28–43)				
18–25	72 (1.9)	1.0	–	1.0	–
26–35	139 (2.0)	1.1 (0.8–1.4)	0.562	1.1 (0.9–1.5)	0.392
36–45	109 (2.4)	1.3 (1.0–1.8)	0.089	1.4 (1.0–1.8)	0.048
46–55	67 (2.9)	1.6 (1.2–2.2)	0.008	1.6 (1.2–2.3)	0.005
>55	8 (6.9)	3.9 (1.8–8.4)	0.0004	4.1 (1.9–8.8)	0.0003
ABO blood type			0.120		
A	148 (2.6)	1.0	–		
B	97 (2.2)	0.8 (0.6–1.1)	0.152		
O	112 (1.9)	0.7 (0.6–0.9)	0.007		
AB	38 (2.3)	0.9 (0.6–1.2)	0.429		
Missing data	0 (0)	–	0.998		
Rh(D) blood type			0.725		
+	390 (2.2)	1.0	–		
−	5 (3.2)	1.4 (0.6–3.5)	0.423		
Missing data	0 (0)	–	0.998		
Ethnicity			0.861		
Han	382 (2.2)	1.0	–		
Non-Han	10 (1.9)	0.8 (0.4–1.6)	0.585		
Missing data	3 (2.2)	1.0 (0.3–3.1)	0.995		
Occupation			0.443		
Healthcare worker	7 (1.6)	1.0	–		
Civil worker	8 (2.2)	1.4 (0.5–3.9)	0.513		
Teacher	19 (3.9)	2.5 (1.0–6.0)	0.042		
Student	30 (2.1)	1.4 (0.6–3.0)	0.542		
Office worker	93 (2.0)	1.3 (0.6–2.8)	0.536		
Worker	34 (2.0)	1.3 (0.5–2.8)	0.611		
Military personnel	2 (2.0)	1.2 (0.3–6.1)	0.787		
Farmer	9 (2.5)	1.6 (0.6–4.4)	0.349		
Others	163 (2.4)	1.5 (0.7–3.2)	0.311		
Missing data	30 (2.2)	1.4 (0.6–3.2)	0.442		
Education level			0.582		
Master/doctorate	15 (1.5)	1.0	–		
Bachelor	113 (2.1)	1.4 (0.8–2.4)	0.247		
College	109 (2.3)	1.5 (0.9–2.6)	0.156		
High school	71 (2.4)	1.6 (0.9–2.7)	0.126		
Lower than high school	38 (2.6)	1.7 (0.9–3.1)	0.082		
Missing data	49 (2.1)	1.4 (0.8–2.5)	0.271		

The association between demographic characteristic and specific antibodies against SARS-CoV-2 were estimated by univariate and multivariate logistic regression.

*OR* odds ratio.

## Discussion

In this study, we systematically investigated the SARS-CoV-2 seroprevalence among large cohorts of healthy blood donors from different cities in mainland China. Our data clearly demonstrated an age- and sex-adjusted SARS-CoV-2 seroprevalence of 2.66% (95% CI: 2.24–3.07%) in Wuhan, 0.033% (95% CI: 0.0029–0.267%) in Shenzhen, and 0.0028% (95% CI: 0.0001–0.158%) in Shijiazhuang, respectively. Although Wuhan is the earliest COVID-19-affected city and had most of the reported COVID-19 patients in China, the SARS-CoV-2 antibody presence was only noted in 2.22% of 17,794 blood donors (2.66% after age–sex standardization) during January–April 2020. Our results suggested that most of the populations of Wuhan remained uninfected during the early wave of COVID-19. Effective blocked SARS-CoV-2 spread in China was also evidenced by the extremely low antibody prevalence among donors in Shenzhen and Shijiazhuang.

More importantly, in tracking antibody presence among Wuhan’s donors in mid-to-late January, there was no confirmatory positivity among 1385 donors from January 15 to 18. In contrast, only one positive donor from 779 donors was found during January 19–22 (donated on January 20, Fig. 2). Given a TAb seroconversion window of 10 days, the earliest emergence of SARS-CoV-2 in Wuhan’s donors should not be earlier than January 10, 2020. The seroprevalence in Wuhan quickly reached the peak (5.21%) about 3 weeks after the first emergence (at the 7th week of 2020 from February 9 to 15), but it subsequently stopped increasing during the city quarantine period (from January 23 to April 7). Interestingly, we detected a decreased adjusted seroprevalence of 2.71% after the lockdown easing (from April 8 to April 30, Fig. 2). According to the newly reported data in Iceland, Canada, China, and the United States, the anti-spike SARS-CoV-2 antibody usually could persist for at least 3–5 months^18–21^. In particular, a new study based on a large cohort of Iceland, which used the same immunoassay kit (Wantai TAb ELISA) as our study, demonstrated that the SARS-CoV-2 antibody did not decline significantly within four months after diagnosis. The reduced seroprevalence among Wuhan’s donors after the lockdown easing may not be attributed to the antibody waning. On the other hand, no significant age or gender difference was noted between donor populations during the Wuhan quarantine period and the stage after easing restrictions in the city (Fig. S1 in the Supplementary Information). Notably, after the lockdown easing, people from nonepidemic areas who returned to work and donated their blood in Wuhan may contribute to the reduced seroprevalence. Although we could not accurately know who returned to Wuhan, the proportion of registered Wuhan residents among the enrolled donors declined from 48.56% (2713/5587) to 39.24% (3941/10,043) after restrictions were lifted, which may serve as evidence to support this explanation. Nevertheless, it was clear that the SARS-CoV-2 seroprevalence ceased to increase in blood donors of Wuhan.

Population-based serological surveillance is a critical approach to assess the prevalence of SARS-CoV-2, as well as to estimate herd immunity. Antibody tests with reliable performance are essential both for clinical diagnosis and epidemiological studies. Our study utilized a TAb-based screening strategy followed by a SARS-CoV-2 pseudovirus-based neutralization test for final confirmation of the antibody presence (Fig. 1). The TAb assay used in this study is a double-sandwich ELISA form, which enables simultaneous detections of IgM, IgG, and IgA against RBD antigen of SARS-CoV-2. Several studies based on cross-assay comparisons in parallel demonstrated that the TAb ELISA outperformed all other assays that only detected single- antibody isotype. However, as the reported specificities of TAb ELISA were ranging from 99.3% to 100%^22,23^, additional neutralization confirmation should be performed, in particular for reactive samples from RNA-negative asymptomatic individuals. In this study, we determined the neutralization activities of all TAb-positive samples against lentiviral-based pseudotyping SARS-CoV-2 virus as the confirmatory approach. The HIV/lentiviral vectors were widely used to produce pseudotyping viral particles bearing various highly pathogenic viral envelopes. As all TAb-positive blood donors in our study were HIV-free, it was easy to exclude the possible interference derived from potential antibodies against surrogate virus proteins, which may be present in samples of some uncertain individuals. Our study revealed that the PPV value of TAb-based screening was highly dependent on the incidence of confirmed COVID-19 cases among different populations. Referring to neutralization results, the false-positive ratio of the TAb-based screening strategy was 0.38% (68/17,794) in Wuhan, 0.37% (25/6810) in Shenzhen, and 0.39% (53/13,540) in Shijiazhuang, without significant difference. However, the PPV values varied from 1.85% (Shijiazhuang) to 85.31% (Wuhan). We noted a TAb/IgG-RBD combining strategy that improved the PPV value from 85.31% to 98.71% with little missing (3.04%, 12/395) of the true-positive cases. However, its performance was still unsatisfactory in donors of Shenzhen and Shijiazhuang (Table 2). Therefore, appropriate tests are required to exclude the false-positive reaction derived from immunoassays that may overestimate the real infection status, particularly for serological studies in a low-prevalence area.

In COVID-19 patients, gender, age, and ABO blood type were reported to be associated with the occurrence or the development of the disease. Compared with females, male patients have higher mortality (22.2% vs. 10.4%) and require longer hospitalization time^24^. A study based on 72,314 COVID-19 patients in China found that the gender ratio in Wuhan’s patients was 0.99 (male/female), but the case fatality rate in males was about 1.64-fold higher than that in females^25^, suggesting an enhanced disease severity for males. The gender difference could be attributed to estrogen receptor signaling-mediated protections, which had been demonstrated in previous SARS-CoV animal study^26^. On the other hand, men smoke more than women in China, and smoking may accelerate lung injury and also be associated with a worse clinical outcome of SARS-CoV-2-related disease^27^. Our study revealed that the SARS-CoV-2 seroprevalence was significantly higher among females than males in healthy donors from Wuhan (adjusted OR: 1.8, 95% CI: 1.5–2.2, Table 3). During the review process of our paper, other two serosurvey studies based on the health population also reported that females had a higher seroprevalence than males^28,29^. Although both females and males are SARS-CoV-2-susceptible, it was possible that females had more probability of developing asymptomatic infections, whereas males are more likely to suffer symptomatic disease after infection. Different genders may also have differences in occupational risk. Another possibility is that females may have more risk of being exposed to the virus, as females may have more responsibilities in caring for sick family members. Consistent with recently reported studies, our data suggested that increasing age was an independent risk factor for SARS-CoV-2 seropositivity (Table 3)^28–30^. Seropositive individuals with older age showed significantly higher titers of IgG-N (Fig. 4b) and neutralization antibodies (Fig. 4c). The age-dependent increased risk was also noted in both COVID-19 patients and asymptomatic infections^31,32^. Age-dependent illness severity may be attributed to immunosenescence and age-related comorbidities. The increased seroprevalence with age suggested an age-dependent effect in the SARS-CoV-2 transmission^30^. The apparent increased risk in older-age population was possibily associated with the less willingness of mask-wearing protection of older people during the early wave of COVID-19. Besides, as the first COVID-19 outbreak occurred on the earlier Spring Festival in China, older adults had more chances for family gatherings, which may increase the virus exposure risk. Moreover, recent studies suggested that the individuals with blood type A showed higher COVID-19 risk, whereas the blood type O was a possible protective factor^33,34^. Our study also noted a lower seroprevalence of SARS-CoV-2 in Wuhan’s donors with blood group O than those with blood type group A in univariate analysis (Table 3). However, the difference was not statistically significant in the logistic regression model. One of the possible reasons is human circulating anti-A antibodies could inhibit the viral adhesion to an ACE2- expressing cell line via blocking the interaction between the virus and its receptor^34,35^. Overall, female and older age were the predominant risk factors independently associated with the seropositivity of SARS-CoV-2.

There are some limitations to our study that should be noted. First, the seroprevalence of our study was derived from data among healthy blood donors, which is a specific population that may have different demographic characteristics to the general population. Although we have standardized the involved donors with the population distribution in the three cities, the “healthy donor effect” should be considered, as people with mild illness or discomfort are not included^36^. Second, due to the limitation of the study population, the seroprevalence of children, teenagers, or the old people (aged > =60 years old) was unable to be estimated from this study. Third, since we did not follow all the confirmed positive donors, and have not obtained their detailed information after donation and the nucleic acid testing results of their respiratory tract samples, the possible exposure way and infection status were unknown.

In summary, from January to April of 2020, the age–sex-standardized prevalence of antibody against SARS-CoV-2 was 2.66% in Wuhan, 0.033% in Shenzhen, and 0.0028% in Shijiazhuang, which was highly associated with the reported COVID-19 case numbers in these cities. The earliest emergence of SARS-CoV-2 seropositivity among blood donors in Wuhan was identified on January 20, 2020, and the overall seroprevalence in this population showed a downward trend from February to April. Moreover, our study provided a prevalence-dependent antibody testing strategy for population-based serological studies, which highlighted the importance of a confirmatory neutralization test in avoiding the misleading of false-positive results of single immunoassay, in particular for a population in the nonepidemic region.

## Methods

### Study design and participants

Blood donors donated from January to April in Wuhan, Shenzhen, and Shijiazhuang were enrolled in the study. The population size, number of confirmed COVID-19 cases, and donation time of enrolled blood donors in three different cities are listed in Table 4. The criteria used for all blood donors during the pandemic are listed as enclosed: (1) all donors should meet the basic requirements of blood donation in China. The legal age for blood donation in China is from 18 years old to 55 years old. If regular donors pass the physical examination, they could donate until 60 years old, or even 65 years old in Shenzhen, (2) have neither a fever (body temperature ≤37.3 °C) nor any respiratory symptoms for at least 28 days, (3) have neither close contact to those confirmed or suspected COVID-19 cases or clustering occurrence of cases for at least 28 days, (4) body temperature is normal before donation (≤37.3 °C), and (5) in Shenzhen and Shijiazhuang, donors who have a history of residence in or travel from Hubei province or have close contact to people from Hubei are suggested to defer blood donation for at least 28 days. A total of 38,144 blood donors were enrolled in this study. Anonymous personal demographic information, including gender, age, ethnicity, occupation and educational level, and blood type was collected.

**Table 4:** The population size, number of confirmed COVID-19 cases and donation time of enrolled blood donors in three different cities.

Blood Center	City	Population^a^ (million)	Number of confirmed COVID-19 cases^b^ (prevalence /100,000)	Donation time of enrolled blood donors
Wuhan Blood Center	Wuhan	11.21	50,333 (448.92)	Jan 15–Apr 30
Shenzhen Blood Center	Shenzhen	13.03	420 (3.22)	Feb 2–Mar 15
Hebei Province Blood Center	Shijiazhuang	11.03	29 (0.26)	Jan 23–Mar 15

^a^The population data was collected from the statistics released by the Hubei Provincial, Shenzhen, and Hebei Provincial Bureau of statistics in 2019.

^b^Data were collected from the website of the Hubei Province Health Committee, Shenzhen Health Committee, and Hebei Province Health Committee.

### Serological tests

After routine tests for transfusion-transmitted pathogens (including HIV antibody), all donation samples were tested for total antibody (TAb) against SARS-CoV-2, and the reactive samples were further tested for SARS-CoV-2-specific IgG and IgM antibodies. All the serologic screening tests used enzyme-linked immunosorbent assay (ELISA) kits provided by Beijing Wantai Biological Pharmacy Enterprise Co., Ltd. The detection experiments were performed according to the manufacturer’s instructions. In brief, TAb detection was based on a double-antigen sandwich immunoassay, using two kinds of mammalian cell-expressed recombinant antigens containing the receptor-binding domain (RBD) of the spike protein of SARS-CoV-2 as the immobilized and HRP-conjugated antigen, respectively. Two kinds of specific IgG antibodies were tested using an indirect ELISA method based on recombinant antigens, RBD antigen (IgG-RBD) and nucleoprotein (IgG-N), respectively. The IgM μ-chain capture method was used to detect the IgM antibodies, using the same HRP-conjugate RBD antigen. Those IgG- or IgM-positive samples with the signal to the cutoff ratio (S/CO) ≥ 10 were further diluted (1:10, 1:40, 1:160…, and 1:40960) by normal saline and tested again. The titer for IgG and IgM antibodies was calculated via S/CO multiplied by the maximum dilution factor.

### Pseudotype lentivirus-based neutralization test (ppNAT)

For confirmation of the presence of neutralizing antibodies, all the TAb-positive donation samples were tested against lentiviral pseudotyping particle (LVpp) bearing SARS-CoV-2 spike antigen. The production and detailed information regarding the assay were described in Supplementary Methods in Supplementary Information. All samples were tested in serial dilutions following the procedure illustrated in Fig. S2 in the Supplementary Information, and the pseudotyping particle-based neutralization titer (ppNAT) of each sample, which is defined as the maximum dilution fold required to achieve infection inhibition by 50% (ID50), was determined by the 4-parameter logistic (4PL) regression. An ID50 ≥ 20 was determined as a cutoff value for the presence of neutralizing antibodies. Due to the limited volume of donation samples, all the serological tests and confirmatory tests were performed once. The sensitivity and specificity of the ppNAT assay were 100% (95% CI: 95.19–100%) and 100% (95% CI: 98.12–100%) based on the evaluation on COVID-19-convalescent samples (*n* = 76) and healthy blood donor samples (*n* = 200, collected before the pandemic outbreak, Fig. S3 in the Supplementary Information).

### Statistical analysis

Crude and age–sex-adjusted seroprevalence were both calculated. Age–sex-adjusted seroprevalence of each city was estimated using the 6th China nationwide population census of each town in 2010 as the standard population. The 95% confidence intervals for age–sex-adjusted rates were calculated using Poisson or Gamma distribution (when the number of cases is minimal). The association between demographic characteristics and specific antibodies against SARS-CoV-2 was estimated by univariate and multivariate logistic regression. Mann–Whitney *U* test was used to compare antibody levels between male and female donors, and Kruskal–Wallis test was used to compare antibody levels among different age groups. Spearman rank correlations were calculated to explore the correlation of ppNAT titer with IgG-RBD, IgG-N, and IgM titers. A *p* value less than 0.05 was considered statistically significant. All the data were collected via Ms Excel 2013 v15.0 (Microsoft Corporation by Impressa Systems, Santa Rosa, California) and these statistical analyses above were realized by SPSS v21.0 (IBM SPSS, Chicago, IL). For donors who provided more than one donation during the study, only one sample was chosen from each donor using simple random sampling by SAS v9.4 (SAS Institute, Cary, NC). All figures were drawn by GraphPad Prism v8.0 (GraphPad Software, San Diego, CA) or Origin 2020 (OriginLab, Northampton, MA).

### Ethical approval

This study was conformed to the ethical guidelines of the 1975 Declaration of Helsinki and was reviewed and approved by the Medical Ethical Committee of Beijing Hospital (2020BJYYEC-070-01). Written informed consent was obtained from each enrolled donor before donation.

### Reporting summary

Further information on research design is available in the Nature Research Reporting Summary linked to this article.

## Supplementary information

Supplementary Information

Reporting Summary

## Data Availability

Source data are provided with this paper. Other related data generated during the current study are available from the corresponding author on reasonable request.

## Source data

Source Data

## References

[1] World Health Organization. WHO Director-General’s opening remarks at the media briefing on COVID-19—11 March 2020. https://www.who.int/director-general/speeches/detail/who-director-general-s-opening-remarks-at-the-media-briefing-on-covid-19---11-march-2020 (2020).

[2] World Health Organization. Coronavirus disease (COVID-19) outbreak situation 2020. https://www.who.int/emergencies/diseases/novel-coronavirus-2019 (2020).

[3] Zhu, N., et al. A novel coronavirus from patients with pneumonia in China, 2019. *N. Engl. J. Med*. 10.1056/NEJMoa2001017 (2020). 10.1056/NEJMoa2001017 PMC7092803 31978945

[4] Wu F (2020). A new coronavirus associated with human respiratory disease in China. Nature.

[5] Wang, P., et al. The SARS-CoV-2 outbreak: diagnosis, infection prevention, and public perception. *Clin Chem*, 10.1093/clinchem/hvaa080 (2020). 10.1093/clinchem/hvaa080 PMC7108508 32154877

[6] Azkur AK (2020). Immune response to SARS-CoV-2 and mechanisms of immunopathological changes in COVID-19. Allergy.

[7] Bai Y (2020). Presumed asymptomatic carrier transmission of COVID-19. JAMA.

[8] Wang Y (2020). Characterization of an asymptomatic cohort of SARS-COV-2 infected individuals outside of Wuhan, China. Clin. Infect. Dis..

[9] Okba NMA (2020). Severe acute respiratory syndrome coronavirus 2-specific antibody responses in coronavirus disease 2019 patients. Emerg. Infect. Dis..

[10] Theel ES (2020). The role of antibody testing for SARS-CoV-2: is there one?. J. Clin. Microbiol..

[11] Zhao J (2020). Antibody responses to SARS-CoV-2 in patients of novel coronavirus disease 2019. Clin. Infect. Dis..

[12] Sethuraman N, Jeremiah SS, Ryo A (2020). Interpreting diagnostic tests for SARS-CoV-2. Jama.

[13] Long QX (2020). Antibody responses to SARS-CoV-2 in patients with COVID-19. Nat. Med..

[14] Wu, F. et al. Neutralizing antibody responses to SARS-CoV-2 in a COVID-19 recovered patient cohort and their implications. *medRxiv*. Preprint at 10.1101/2020.03.30.20047365 (2020).

[15] Fontanet, A., et al. Cluster of COVID-19 in northern France: a retrospective closed cohort study. *medRxiv.* Preprint at 10.1101/2020.04.18.20071134 (2020).

[16] Korth J (2020). SARS-CoV-2-specific antibody detection in healthcare workers in Germany with direct contact to COVID-19 patients. J. Clin. Virol..

[17] Sood N (2020). Seroprevalence of SARS-CoV-2–specific antibodies among adults in Los Angeles County, California, on April 10-11, 2020. JAMA.

[18] Gudbjartsson DF (2020). Humoral immune response to SARS-CoV-2 in Iceland. N. Eng. J. Med..

[19] Isho B (2020). Persistence of serum and saliva antibody responses to SARS-CoV-2 spike antigens in COVID-19 patients. Sci. Immunol..

[20] Tan, Y. et al. Durability of neutralizing antibodies and T-cell response post SARS-CoV-2 infection. *Front. Med*. **14**, 746–751 (2020). 10.1007/s11684-020-0822-5 PMC7533664 33017040

[21] Wajnberg A (2020). Robust neutralizing antibodies to SARS-CoV-2 infection persist for months. Science.

[22] GeurtsvanKessel, C. H., et al. Towards the next phase: evaluation of serological assays for diagnostics and exposure assessment. *medRxiv.* Preprint at 10.1101/2020.04.23.20077156 (2020).

[23] Lassaunière, R., et al. Evaluation of nine commercial SARS-CoV-2 immunoassays. medRxiv. Preprint at 10.1101/2020.04.09.20056325 (2020).

[24] Qin L (2020). Gendered effects on inflammation reaction and outcome of COVID-19 patients in Wuhan. J. Med. Virol..

[25] Epidemiology Working Group for NCIP Epidemic Response. (2020). The epidemiological characteristics of an outbreak of 2019 novel coronavirus diseases (COVID-19) in China. Chin. J. Epidemiol..

[26] Channappanavar R (2017). Sex-based differences in susceptibility to severe acute respiratory syndrome coronavirus infection. J. Immunol..

[27] Di Stadio A, Della Volpe A, Ralli M, Ricci G (2020). Gender differences in COVID-19 infection. The estrogen effect on upper and lower airways. Can it help to figure out a treatment?. Eur. Rev. Med. Pharmacol. Sci..

[28] Pan, Y., et al. Seroprevalence of SARS-CoV-2 immunoglobulin antibodies in Wuhan, China: part of the city-wide massive testing campaign. *Clin. Microbiol. Infect*. 10.1016/j.cmi.2020.09.044 (2020). 10.1016/j.cmi.2020.09.044 PMC7539137 33035672

[29] Liu A (2020). Seropositive prevalence of antibodies against SARS-CoV-2 in Wuhan, China. JAMA Netw. Open.

[30] Davies NG (2020). Age-dependent effects in the transmission and control of COVID-19 epidemics. Nat. Med..

[31] Chen N (2020). Epidemiological and clinical characteristics of 99 cases of 2019 novel coronavirus pneumonia in Wuhan, China: a descriptive study. Lancet.

[32] Huang C (2020). Clinical features of patients infected with 2019 novel coronavirus in Wuhan, China. Lancet.

[33] Zhao, J., et al. Relationship between the ABO Blood Group and the COVID-19 Susceptibility. *Clin. Microbiol. Infect*. 10.1093/cid/ciaa1150 (2020).

[34] Li J (2020). Association between ABO blood groups and risk of SARS-CoV-2 pneumonia. Br. J. Haematol..

[35] Gérard C, Maggipinto G, Minon JM (2020). COVID-19 & ABO blood group: another viewpoint. Br. J. Haematol..

[36] Atsma F, Veldhuizen I, Verbeek A, de Kort W, de Vegt F (2011). Healthy donor effect: its magnitude in health research among blood donors. Transfusion.

