# Supplementary material for: The prevalence of antibodies to SARS-CoV-2 among blood donors in China
Source: Nat Commun. 2021 Mar 2;12:1383. doi: 10.1038/s41467-021-21503-x (PMC7925561; doi:10.1038/s41467-021-21503-x)

Supplementary Information

The prevalence of antibodies to SARS-CoV-2 among blood donors in China

Le Chang, Wangheng Hou, Lei Zhao, Yali Zhang, Yanbin Wang, Linfeng Wu, Tingting Xu, Lilin Wang, Juan Wang, Jian Ma, Lan Wang, Junpeng Zhao, Jing Xu, Juan Dong, Ying Yan, Ru Yang, Yu Li, Fei Guo, Wenjuan Cheng, Yingying Su, Jinfeng Zeng, Wei Han, Tong Cheng, Jun Zhang, Quan Yuan, Ningshao Xia, Lunan Wang

Supplementary Methods

Neutralization tests of plasma samples against lentiviral pseudotyping particle (LVpp) bearing SARS-CoV-2 spike.

Briefly, SARS-CoV-2 LVpp were generated by cotransfection of a lentiviral packaging plasmid (psPAX2, Addgene#12260), a SARS-CoV-2 spike expression plasmid (containing codon-optimized spike gene derived from the strain of MN908947.3) and a green fluorescent protein (mNeonGreen) reporter vector (pLvEF1α-mNG, carrying EF1α promoter-driven mNeonGreen expressing cassette) in 293T cells. Infection and neutralization assays were performed on H1299-ACE2hR cells, which stably over-expressed human ACE2 (enabling it is highly susceptible to SARS-CoV-2 virus) and nuclear-localized RFP (H2B-mRuby3, allowing accurate cell counting) based on H1299 cells. For ppNAT tests, serially-diluted plasma samples (1:10, 1: 40, 1:160, 1:640, 1:2560, 1:10240) were incubated with LVpp inoculum (0.5 TU/cell) for 1 hour. Subsequently, the mixtures were incubated with the cells, which had been pre-seeded in 96-well cell culture plates with an optically-clear bottom. After 36-hour incubation, the plates were imaged by using Opera Phenix or Operetta CLS high-content equipment (PerkinElmer). For quantitative determination, fluorescence images were analyzed by Columbus Software 2.5.0 (PerkinElmer), the numbers of mNeonGreen(+) cells per well were calculated to indicate the infection performance, and the total cell numbers per well were also counted to normalize the readouts. The reduction (%) on mNeonGreen(+) cells of the plasma-treated well in comparison with control-well was calculated to show the neutralization activity. The ppNAT titer of each samples were expressed as the maximum dilution fold required to achieve infection inhibition by 50% (ID50). The ID50 value was based on inhibition ratio of serial dilutions and was determined by the 4-parameter logistic (4PL) regression using GraphPad Prism v8.0.

Supplementary Figures

Figure S1. Distribution of donors in three different stages in Wuhan.

a Age and sex distribution among the three different stages (pre-lockdown, quarantine, and lifting restrictions) in Wuhan. **b** Proportion of different sexes in the three stages. The proportion of female donors before lockdown stage was lower than that after lockdown ($p<0.001$), but the proportion of female donors between the quarantine stage and unsealing stage showed no difference ($p>0.05$). **c** Proportion of different age groups in the three stages. The proportions of age group 26-35, 36-45, and 46-55 in the quarantine stage was higher than the other two stages ($p<0.001$). p-values by Pearson's chi-squared test followed by Bonferroni's multiple comparisons post hoc test (two-sided) are indicated in **b** and **c**.

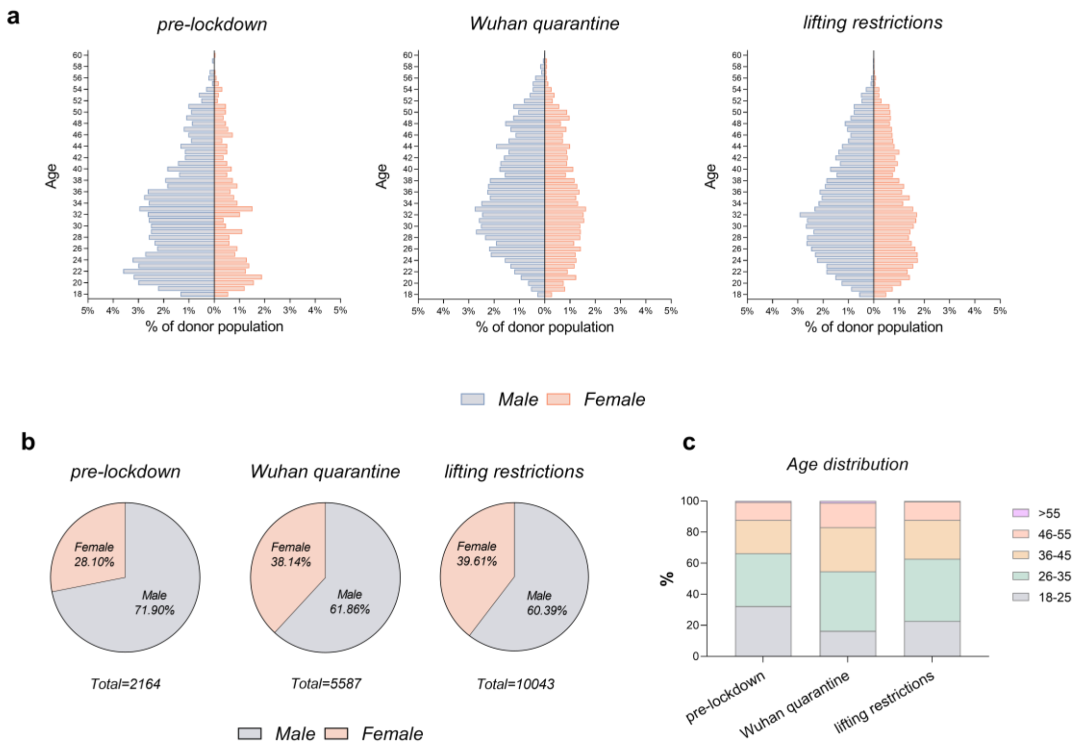

Figure S2. An illustration of the determinations of neutralization activity against SARS-CoV-2 pseudotyping virus for blood samples.

The detailed detection procedure of LVpp-based neutralization tests (LVppNAT) in determining of the neutralization activities of blood samples was described in the Methods section. This picture showed an example of the LVppNAT measurements of 9 samples (WH10-WH18) on H1299-ACE2hR cell cultures. The infected-cells were green fluorescence-activated due to the expression of the mNeonGreen reporter, whereas the cell nucleus was visualized by H2B-mRuby3 (mRuby3 is a red fluorescent protein). For each well, a total of 25 view fields were imaged (scale bar, 500μm), and the total mNeonGreen(+) cells were counted to calculate the ID50 (maximal dilution ratio at half infection inhibition) in referring to virus-only control wells. Due to the limited volume of blood samples, all the LVppNAT were performed only once. The grey dotted lines show the value of half infection inhibition.

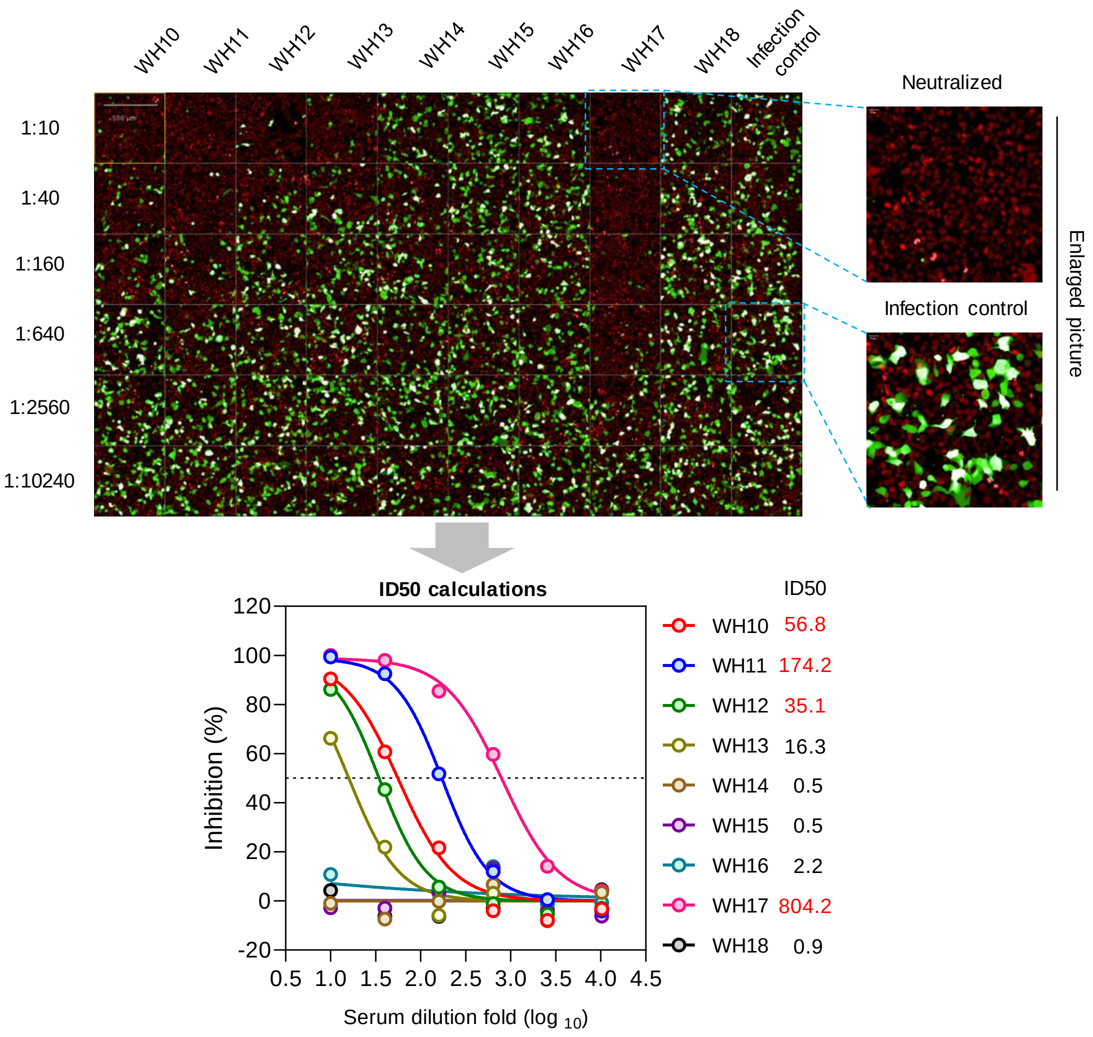

Figure S3. The diagnostic performance of the pseudotype lentivirus-based neutralization tests (ppNAT).

To evaluate the sensitivity and specificity of the pseudotype lentivirus-based neutralization tests (ppNAT) assay, plasma samples from 76 confirmed COVID-19 patients (collected in the convalescent phase and at day 17-94 since illness onset) and 200 healthy blood donors (these samples were collected in July 2019 before the COVID-19 outbreak) were tested. None of the healthy donor samples showed detectable ppNAT reactivity (ID50 ranging from 0.5-15.6), whereas all COVID-19-convalescent ones were positive (ID50 ranging from 52-19,340). Therefore, the sensitivity and specificity of the ppNAT assay was 100% (95%CI: 95.19%-100%) and 100% (95%CI: 98.12%-100%). A dotted line indicates the cutoff value (ID50=20) and the error bar represents one standard deviation from the mean.

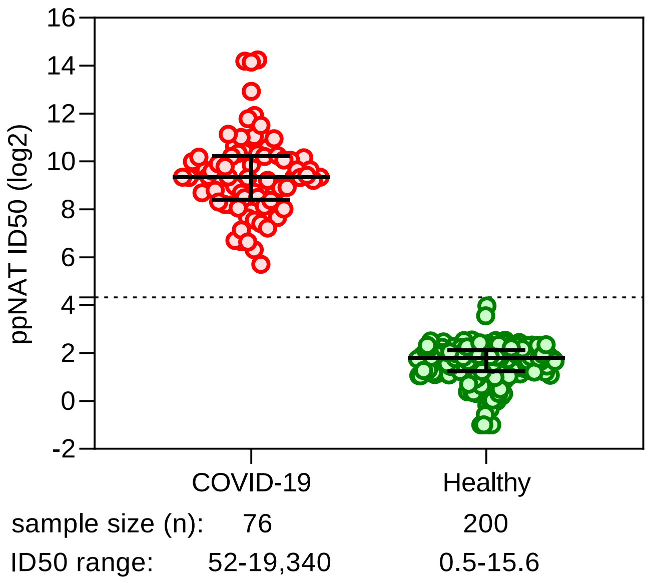

Supplement: Supplementary file 1 — Supplementary Information [file 41467_2021_21503_MOESM1_ESM.pdf]
