# Supplementary material for: The prevalence of antibodies to SARS-CoV-2 among blood donors in China
Source: Nat Commun. 2021 Mar 2;12:1383. doi: 10.1038/s41467-021-21503-x (PMC7925561; doi:10.1038/s41467-021-21503-x)
Supplement: Supplementary file 2 — Reporting Summary [file 41467_2021_21503_MOESM2_ESM.pdf]

nature research

Corresponding author(s): Quan Yuan and Lunan Wang

Last updated by author(s): Dec 13, 2020

Reporting Summary

Nature Research wishes to improve the reproducibility of the work that we publish. This form provides structure for consistency and transparency in reporting. For further information on Nature Research policies, see our Editorial Policies and the Editorial Policy Checklist.

Statistics

For all statistical analyses, confirm that the following items are present in the figure legend, table legend, main text, or Methods section.

n/a	Confirmed	
☐	☒	The exact sample size (n) for each experimental group/condition, given as a discrete number and unit of measurement
☐	☒	A statement on whether measurements were taken from distinct samples or whether the same sample was measured repeatedly
☐	☒	The statistical test(s) used AND whether they are one- or two-sided<br>*Only common tests should be described solely by name; describe more complex techniques in the Methods section.*
☐	☒	A description of all covariates tested
☐	☒	A description of any assumptions or corrections, such as tests of normality and adjustment for multiple comparisons
☐	☒	A full description of the statistical parameters including central tendency (e.g. means) or other basic estimates (e.g. regression coefficient) AND variation (e.g. standard deviation) or associated estimates of uncertainty (e.g. confidence intervals)
☐	☒	For null hypothesis testing, the test statistic (e.g. F, t, r) with confidence intervals, effect sizes, degrees of freedom and P value noted<br>*Give P values as exact values whenever suitable.*
☒	☐	For Bayesian analysis, information on the choice of priors and Markov chain Monte Carlo settings
☒	☐	For hierarchical and complex designs, identification of the appropriate level for tests and full reporting of outcomes
☐	☒	Estimates of effect sizes (e.g. Cohen's d, Pearson's r), indicating how they were calculated

Our web collection on statistics for biologists contains articles on many of the points above.

Software and code

Policy information about availability of computer code

Data collection	Ms Excel 2013, version 15.0
Data analysis	Statistical analyses were realized by SPSS v21.0 (IBM SPSS, Chicago, IL). For donors who provided more than one donation during the study, only one sample was chosen from each donor using simple random sampling by SAS v9.4 (SAS Institute, Cary, NC). All figures were drawn by GraphPad Prism v8.0 (GraphPad Software, San Diego, CA) or Origin 2020 (OriginLab, Northampton, MA).

For manuscripts utilizing custom algorithms or software that are central to the research but not yet described in published literature, software must be made available to editors and reviewers. We strongly encourage code deposition in a community repository (e.g. GitHub). See the Nature Research guidelines for submitting code & software for further information.

Data

Policy information about availability of data

All manuscripts must include a data availability statement. This statement should provide the following information, where applicable:

- Accession codes, unique identifiers, or web links for publicly available datasets
- A list of figures that have associated raw data
- A description of any restrictions on data availability

The source data in Figures are provided with this paper. Other related data generated during the current study are available from the corresponding author on reasonable request.

Field-specific reporting

Please select the one below that is the best fit for your research. If you are not sure, read the appropriate sections before making your selection.

☒ Life sciences ☐ Behavioural & social sciences ☐ Ecological, evolutionary & environmental sciences

For a reference copy of the document with all sections, see nature.com/documents/nr-reporting-summary-flat.pdf

Life sciences study design

All studies must disclose on these points even when the disclosure is negative.

Sample size	No sample size calculation was performed. We involved all available data in the analysis.
Data exclusions	Since there were some donors who provided more than one donation during the study, which may bring potential bias when calculated the seroprevalence of SARS-CoV-2, before we calculated the data,only one sample was chosen from each donor using simple random sampling by SAS v9.4 (SAS Institute, Cary, NC). Other samples from this donor were excluded.
Replication	Due to the limited volume of samples from each donor, all the testings (SARS-CoV-2 total antibody, IgG, and IgM antibody, and the confirmatory tests) were performed only once in the study.
Randomization	Our study is an observation study, and we involved all donors donated their blood during the study. Therefore, the participants were not selected randomly.
Blinding	Antibodies detection were performed automatically by researchers blind to samples information. Data analysis were performed by different trained researchers.

Reporting for specific materials, systems and methods

We require information from authors about some types of materials, experimental systems and methods used in many studies. Here, indicate whether each material, system or method listed is relevant to your study. If you are not sure if a list item applies to your research, read the appropriate section before selecting a response.

Materials & experimental systems

n/a	Involved in the study	
☒	☐	Antibodies
☐	☒	Eukaryotic cell lines
☒	☐	Palaeontology and archaeology
☒	☐	Animals and other organisms
☐	☒	Human research participants
☒	☐	Clinical data
☒	☐	Dual use research of concern

Methods

n/a	Involved in the study	
☒	☐	ChIP-seq
☒	☐	Flow cytometry
☒	☐	MRI-based neuroimaging

Eukaryotic cell lines

Policy information about cell lines

Cell line source(s)	HEK293T, H1299, H1299-ACE2hR. The H1299-ACE2hR which stably over-expressed human ACE2 and and nuclear-localized RFP was constructed by lentiviral transduction based on H1299 cells.
Authentication	The HEK293T and H1299 were purchased from ATCC. H1299-ACE2hR cells were constructed in our laboratory.
Mycoplasma contamination	Negative for mycoplasma
Commonly misidentified lines (See ICLAC register)	No commonly misidentified lines was used.

Human research participants

Policy information about studies involving human research participants

Population characteristics	The characteristics of all involved donors were summarized in Table 2. The median age was 33 (IQR, 19 to 47), 36 (IQR, 19 to 53), and 40 (IQR, 33 to 48) for donors from the three cities, respectively. Among these donors, 29.5% to 37.7% were female.
Recruitment	All the participants were voluntary blood donors who donated blood during the pandemic. All the involved samples were remaining samples after routine testing of transfusion-transmitted pathogens. Since all donors donated blood during the study were involved, there were no self-selection bias. However, due to the limitation of the study population, the seroprevalence may not reflect the true data in the general population and the seroprevalence of children, teenagers, or the old people (aged >=60 years old) were unable to be estimated from this study.
Ethics oversight	This study was reviewed and approved by the Medical Ethical Committee of Beijing Hospital (2020BJYYEC-070-01). Written informed consent was obtained from each enrolled donor before donation.

Note that full information on the approval of the study protocol must also be provided in the manuscript.
